# Electron microscopy studies on interfacial solid-state reactions induced by electronic excitation

**DOI:** 10.1093/jmicro/dfaf029

**Published:** 2025-06-05

**Authors:** Kazuhisa Sato

**Affiliations:** Research Center for Ultra-High Voltage Electron Microscopy, The University of Osaka, 7-1 Mihogaoka, Ibaraki, Osaka 567-0047, Japan

**Keywords:** electronic excitation, electron irradiation, core-hole, Auger decay, solid-state reaction

## Abstract

We have studied the effects of electron irradiation on Pt/a-SiO_*x*_ thin films by transmission electron microscopy and electron diffraction. Pt_2_Si was formed by 75 keV electron irradiation at 298 K and 90 K. Such a low-temperature synthesis of Pt_2_Si can be attributed to the dissociation of a-SiO_*x*_ induced by electronic excitation; Si–O bonds dissociate through Auger decay of core-holes generated by electronic excitation, and then, dissociated Si atoms form Pt–Si bonds. The morphology of Pt islands extensively changed during Pt_2_Si formation, even at 90 K. Coalescence and growth of metallic particles are not due to thermal effects during electron irradiation but to athermal processes accompanied by silicide formation. To maintain the reaction interface between metallic particles and the dissociated Si atoms by electronic excitation, a considerable concomitant morphology change occurs. Similarly, Fe_2_Si was synthesized by using the same technique. In this way, we have demonstrated a versatile method for selectively forming nanoscale metal silicides in electron-irradiated areas at room temperature. We also propose a new mechanism for the crystallization of amorphous alloys, which is mediated by additional solute atoms produced by electronic excitation. Crystallization of amorphous Pd–Si alloy thin films can be realized by 75 keV electron irradiation at 90 K via the electronic excitation, where both knock-on damage and possible thermal crystallization can be excluded. Supply of dissociated Si to the Pd–Si layer may cause instability of the amorphous phase, which serves as the trigger for the remarkable structural change, i.e. additional solute atom-mediated crystallization.

## Teaser

A versatile method for selectively forming nanoscale metal silicides in electron irradiated areas at room temperature or below has been demonstrated using electron microscopy. This method can also be applied to the crystallization of amorphous materials, termed as solute atom mediated crystallization.

## Introduction

High-energy electron irradiation is widely used as an effective method for modifying materials’ microstructures [1 and references therein]. Research on generation, annihilation and migration of point defects due to electron irradiation contributes to elucidating various processes occurring in solids [2,3]. If we focus on nonmetallic inorganic materials, examples of research in this field include the formation of structural defects in Si [4,5], recoil implantation of foreign atoms into semiconductors [6], amorphization of Si [7,8] and crystallization of amorphous Sb nanoparticles [9].

In general, materials modification by high-energy electron irradiation can be achieved by two different routes, namely knock-on atom displacement and electronic excitation [10]. The former process, originating from the Coulomb interaction between a fast incident electron and an atomic nucleus, has acquired a general understanding based on the vast amount of accumulated knowledge so far. On the other hand, our understanding of electronic excitation effects, i.e. inelastic scattering due to Coulomb interaction between an incident electron and the atomic electrons, in relation to materials modification is still insufficient. The latter process involves, in some cases, atom migration triggered by excitation of electrons in a solid. This would lead to a novel method for materials modification if the atom migration can be controlled.

In view of this background, research on structural changes due to electronic excitation can be summarized as follows. First, electronic excitation is divided into excitation of valence electrons and inner-shell electrons (core excitation), and the former is studied mainly using laser irradiation; desorption of constituent atoms from the surface [11,12] and formation of metastable surface nanostructures [13,14] have been reported. A research group has discovered solid-state reactions that are suspected to involve electronic excitation: (i) phase separation of GaSb nanoparticles by 25 keV electron irradiation [15] and (ii) formation of α-Pt_2_Si at the Pt/amorphous (a−) SiO_*x*_ (*x* ∼ 1.5) interface by electron irradiation (25–200 keV) [16]. These solid-state reactions cannot proceed by thermal annealing because the change in Gibbs free energy for each reaction is positive. The thermodynamic constraint strongly suggests that electronic excitation is certainly involved in these solid-state reactions.

**Fig. 1.**
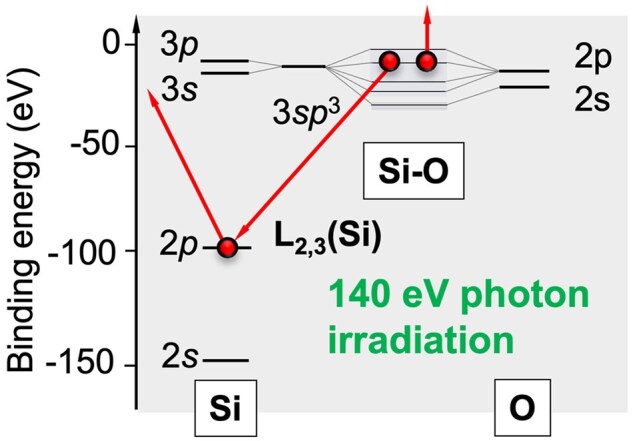
Schematic diagram showing the L_23_VV Auger electron emission after Si 2p core electron excitation by 140 eV photon irradiation.

To clarify the contribution of electronic excitation effects to α-Pt_2_Si formation at the Pt/a-SiO_*x*_ interface, the author of this review and collaborators carried out photon irradiation experiments using synchrotron radiation [17,18]. We found that silicide formation was induced by irradiation with 680 and 140 eV photons but not by irradiation with 80 eV photons. These results indicate that excitation of the valence band electrons only is insufficient to induce silicidation and that at least excitation of core electrons in the Si 2p level (99 eV) is necessary for silicide formation to occur. The core-hole relaxes via the Auger transition. In the final state of the Auger transition, two holes are generated in the valence band, and hence it is inferred that the valence of the oxygen ion changes (O^2−^ → O^0^). Thus, Auger decay of a core-hole (L_23_VV Auger electron emission) may trigger dissociation of a Si–O bond, while it is immediately recovered by recombination. It is also possible for an Si atom that becomes free from the neighboring O atom for a short time to be trapped by a Pt atom at the Pt/a-SiO_*x*_ interface, i.e. the formation of a Pt–Si bond. This mechanism is similar to that of the Knotek–Feibelman (K-F) model [19] for oxygen desorption from transition metal oxide surfaces. By using energy-tunable synchrotron radiation as an excitation source, orbital-selective electronic excitation becomes possible, and we have reached the conclusion mentioned above. A schematic diagram showing the mechanism of Si–O bond breakage is shown in Fig. 1. It should be noted that in the case of electron irradiation using a transmission electron microscopy (TEM; 75 or 200 keV electrons in this study), not only Si2p but all inner-shell electrons can be excited according to their respective ionization excitation cross sections [20].

In this review, we introduce two kinds of examples of solid-state reactions initiated by the dissociation of a-SiO_*x*_ by electronic excitation: (i) formation of metal silicide at metal/a-SiO_*x*_ interfaces [21,22] and (ii) crystallization of an amorphous alloy thin film [23]. In either case, Si content in the matrix decreases after the solid-state reaction, i.e. a-SiO_*x*_ acts as a source of pure Si atoms.

## Experimental procedure

### Specimen preparation

Thin films of Pt and a-SiO_*x*_ (hereafter, Pt/a-SiO_*x*_) were prepared by dc magnetron sputtering of Pt onto an a-SiO_*x*_ film kept at room temperature, which was formed by vapor deposition of silicon monoxide SiO on cleaved NaCl(001) or on Si(111) prior to the sputtering of Pt [21]. The oxygen content, *x*, in the a-SiO_*x*_ film was ∼ 1.5 (SiO_1.5_) as determined in a prior study [16]. Some of the specimens were further coated by a-SiO_*x*_ to prepare Pt islands embedded between a-SiO_*x*_ films (i.e. sandwich-type a-SiO_*x*_/Pt/a-SiO_*x*_). The substrate temperature was kept at room temperature during the deposition. Similarly, a-SiO_*x*_/Fe/a-SiO_*x*_ thin films were prepared by evaporating Fe instead of Pt [22].

Thin films of a-Pd–Si alloy were fabricated by the co-deposition of Pd and Si targets using dc magnetron sputtering [23]. Two kinds of substrates, NaCl(001) cleaved in air and a-SiO_*x*_ thin films deposited on NaCl substrates, were used. The substrate temperature was kept at room temperature during the sputtering. Sputtering was performed in high-purity Ar (99.999%) gas at a pressure of 8 Pa and a power of 100 W. A part of the specimen film was peeled off from the NaCl substrate and transferred onto a Si(111) substrate.

The as-deposited films prepared on the NaCl substrates were removed by immersing the substrate into distilled water, and floated specimen films were mounted onto copper grids for TEM observation (plan-view observation). Cross-sectional TEM specimens were prepared from the films deposited or mounted on the Si(111) substrates using a focused ion beam (FIB) instrument (Thermo Fisher Scientific Scios2 Dual Beam) (cross-sectional view observation).

### Electron irradiation and TEM observation

The prepared thin films on copper grids were irradiated with 75 keV electrons using a TEM (Hitachi H-7000 with a LaB_6_ cathode). The electron dose rate (electron flux) was estimated using a Faraday cage attached to the TEM. Dose rates used for the electron irradiation experiments were in the range between 7.5 × 10^22^ electrons/m^2^s (hereafter, e/m^2^s) and 3.0 × 10^24^ e/m^2^s. Irradiation was carried out at 298 and 90 K. Structural changes by electron irradiation were observed *in situ* using the 75 kV-TEM. The observations were carried out at dose rates one order of magnitude lower than those used for electron irradiation experiments. High-resolution TEM (HRTEM) images were observed using a 200 kV-TEM (JEOL JEM-ARM200F with a Schottky field emission gun). TEM images and selected area electron diffraction (SAED) patterns were recorded using a CCD camera (Gatan Orius200 attached to the 75 kV-TEM) or a CMOS camera (Gatan OneView attached to the 200 kV-TEM). Compositional analysis was performed in scanning mode (Scanning Transmission Electron Microscopy; STEM) using an energy-dispersive x-ray spectrometer (EDS, JEOL JED-2300) attached to the 200 kV-TEM. Specimen thickness was measured by electron energy-loss spectroscopy using a post-column energy filter (Gatan Continium K3).

## α-Pt_2_Si formation at Pt/a-SiO_*x*_ interface [21]

### A brief overview of metal silicide formation

Metal silicides are indispensable contact materials in current Si-based microelectronics technology, and hence much research focuses on their formation mechanism and phase stability [24]. Researchers commonly study transition metal silicides as thermoelectric devices as well [25]. A solid-state reaction of a metallic element with Si is the most popular manufacturing method for metal silicides. State variables (e.g. pressure, temperature, activities of the components) determine the reaction equilibrium, and the Gibbs free energy change informs one as to whether or not a reaction is feasible. In general, Si is highly chemically active; some metallic elements (e.g. Ni, Pd and Pt) that come into contact with pure Si form silicides at relatively low annealing temperatures [26]. All these phenomena correspond to a thermally activated atomic reaction; namely, thermal interfacial reaction between a metallic element and Si [27,28].

The solid-state reaction induced by electronic excitation is another route to promote a metallic silicide, and hence it has potential applications to microfabrication because the metal silicide selectively forms only in the irradiated area. To understand the mechanism of metallic silicide formation induced by electronic excitation, the role of the metal/a-SiO_*x*_ interfacial area must be clarified. In this section, the results of electron irradiation effects on Pt/a-SiO_*x*_ thin films are shown as a model system.

### Electron irradiation at 298 K

**Fig. 2.**
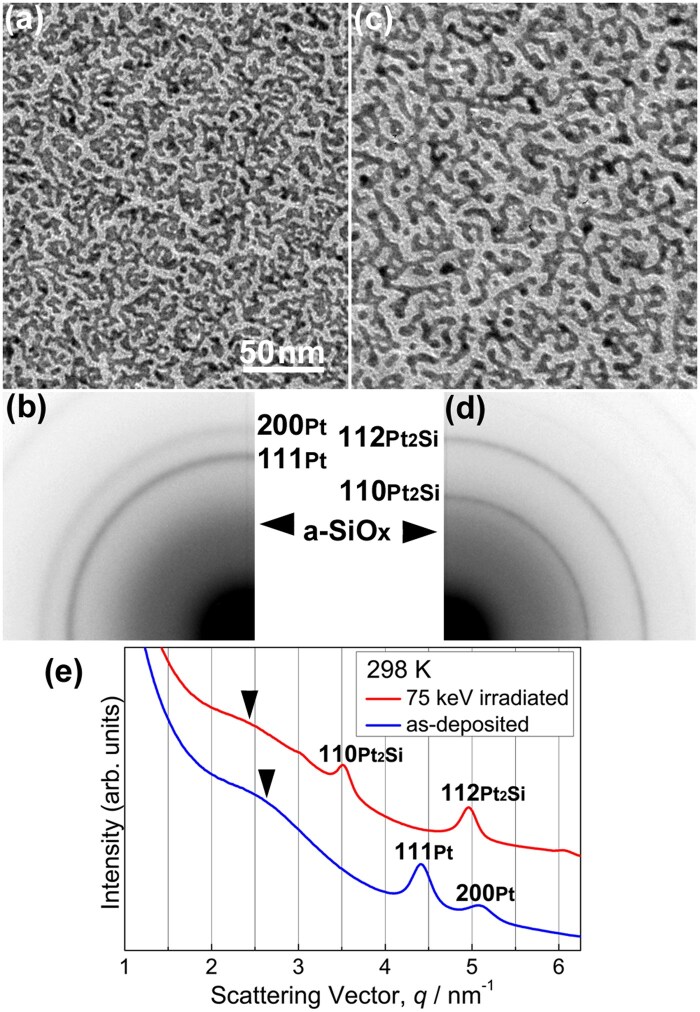
(a and b) BF-TEM images and SAED patterns, respectively, of as-deposited an a-SiO_*x*_/Pt/a-SiO_*x*_ thin film. (c and d) BF-TEM images and SAED patterns, respectively, of an a-SiO_*x*_/Pt/a-SiO_*x*_ thin film, after 75 keV electron irradiation at 298 K for 3.6 ks. (e) Intensity profiles of SAED patterns measured in the radial direction. Reprinted with permission from Sato and Mori [21] under CC-BY-NC-ND 4.0.

**Fig. 3.**
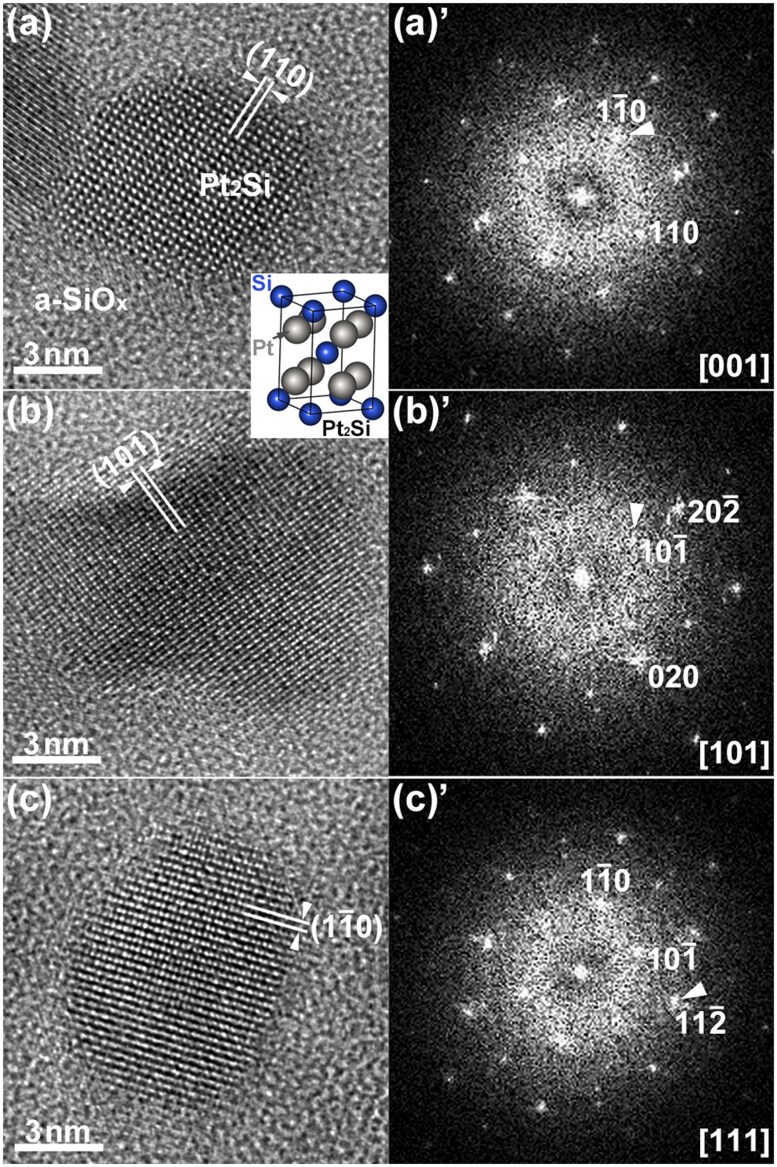
HRTEM images and FFT patterns of the tetragonal α-Pt_2_Si formed by 75 keV electron irradiation at 298 K (total dose: 2.7 × 10^26^ e/m^2^). The beam incidence directions for Pt_2_Si are (a)(a)′ [001], (b)(b)′ [101] and (c)(c)′ [111]. The crystal structure of α-Pt_2_Si is shown in the inset.

Figures 2a and b show a bright-field (BF) TEM image and the corresponding SAED pattern, respectively, of an as-deposited a-SiO_*x*_/Pt/a-SiO_*x*_ thin film. Discontinuous island-like structures of Pt formed in the a-SiO_*x*_ matrix. The SAED pattern is composed of Debye–Scherrer rings of face-centered cubic (fcc) Pt and a halo pattern of a-SiO_*x*_. After 75 keV electron irradiation at 298 K for 3.6 ks, the morphology of the metallic particles (Pt or Pt-Si) considerably changed (Fig. 2c). The electron dose rate was 7.5 × 10^22^ e/m^2^s. Namely, total dose irradiated was 2.7 × 10^26^ e/m^2^. Particle coalescence and growth occurred during electron irradiation. The SAED pattern clearly shows a structural change after electron irradiation; α-Pt_2_Si (ThH_2_-type structure, I4/mmm) is formed (Fig. 2d) (the crystal structure is shown in the inset of Fig. 3). Silicide formation at the Pt/a-SiO_*x*_ interface does not occur thermally as mentioned in the ‘Introduction’ section. It should be noted that under 75 keV electron irradiation, atomic migration induced by knock-on atom displacement can be excluded since the electron energy of 75 keV is below the threshold of knock-on atom displacement both for Pt (1.3 MV) [29] and Si (216 kV or 197 kV for Si in SiO_2_) [30]. Instead, electronic excitation makes it possible to form Pt_2_Si at the Pt/a-SiO_*x*_ interface.

Figure 2e shows intensity profiles measured in the radial direction of the SAED patterns of the specimens before and after electron irradiation (i.e. taken from Fig. 2b and d, respectively). We integrated the intensity in the circumferential direction. Formation of Pt_2_Si after electron irradiation is clearly seen as reflections of such as 110 and 112, and there are no reflections of Pt. Arrowheads indicate the peak position of the first halo ring of the a-SiO_*x*_ film. The peak shift toward lower spatial frequencies occurs after electron irradiation. The peak shift is due to the change in the chemical composition of a-SiO_*x*_ (i.e. increase in oxygen content) associated with Pt_2_Si formation. Si depletion in a-SiO_*x*_ leads to an increase in the oxygen content toward a-SiO_2_. The peak position of the first halo ring for as-deposited a-SiO_*x*_ is 2.60 nm^−1^ and that for a-SiO_2_ is 2.44 nm^−1^ [17].

Figure 3 shows HRTEM images of the α-Pt_2_Si formed by 75 keV electron irradiation at 298 K. Fast Fourier Transform (FFT) patterns of each image are also shown in the right panels. Total dose irradiated was 2.7 × 10^26^ e/m^2^. The Pt_2_Si is embedded in the a-SiO_*x*_ film, and the salt–pepper contrast around the crystalline region is due to the a-SiO_*x*_. By analyzing the crossed lattice fringes and their lattice spacings, the beam incidence directions for crystalline Pt_2_Si were determined as follows: (a)(a)’ [001], (b)(b)′ [101] and (c)(c)’ [111]. The lattice spacings of the (110) and (10$\overset{-}{1}$) of the tetragonal Pt_2_Si are 0.28 nm and 0.33 nm, respectively.

**Fig. 4.**
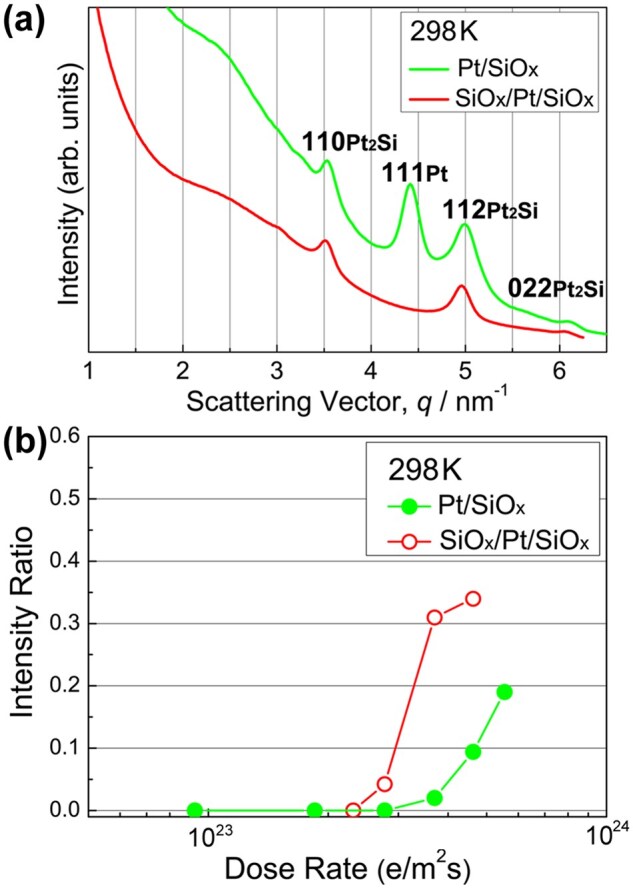
(a) Intensity profiles of the SAED patterns obtained for Pt/a-SiO_*x*_ and a-SiO_*x*_/Pt/a-SiO_*x*_ thin films after 75-keV electron irradiation at 298 K for 3.6 ks. (b) Dose rate dependence of the integrated intensity ratios of 110_Pt2Si_ and 111_Pt_ reflections (*I*_110_/*I*_111_) at 298 K for 75 keV electrons. Reprinted with permission from Sato and Mori [21] under CC-BY-NC-ND 4.0.

Figure 4a compares the intensity profiles of the SAED patterns obtained for the Pt/a-SiO_*x*_ bilayer film and the a-SiO_*x*_/Pt/a-SiO_*x*_ sandwiched film after 75 keV electron irradiation at 298 K for 3.6 ks. The electron dose rate was 7.5 × 10^22^ e/m^2^s in both cases (total dose: 2.7 × 10^26^ e/m^2^). A reflection of 111_Pt_ remains in the Pt/a-SiO_*x*_ bilayer film after electron irradiation, coexisting with newly formed reflections of Pt_2_Si (green line). The presence of unreacted Pt indicates that the formation of Pt_2_Si is still at an intermediate stage. However, Pt reflections were not present in the a-SiO_*x*_/Pt/a-SiO_*x*_ sandwiched film (red line); namely, silicide formation has been completed in this specimen. We estimate the interfacial area between Pt and a-SiO_*x*_ in the a-SiO_*x*_/Pt/a-SiO_*x*_ sandwiched film as roughly twice that of the Pt/a-SiO_*x*_ bilayer film; this interfacial area difference may have served as an essential factor in promoting the interfacial reaction. Assuming that the reaction rate is constant, the total quantity of reaction products proportionally depends on the interfacial area. All these considerations suggest that the extent of the interfacial area may correspond to the progress of Pt_2_Si formation.

Figure 4b shows the dose rate dependence of the integrated intensity ratios of 110_Pt2Si_ and 111_Pt_ reflections (*I*_110_/*I*_111_) of the Pt/a-SiO_*x*_ bilayer film and of the a-SiO_*x*_/Pt/a-SiO_*x*_ sandwiched film. We extracted the intensity ratios from the SAED patterns obtained using 75 keV electrons at 298 K. The irradiation time was 600 s for all the measurements. The dose rate of the order of 10^23^ e/m^2^s is one order higher than that used in Figs. 2–4a. The sandwiched film always shows a rapid increase in intensity ratio compared with the Pt/SiO_*x*_ bilayer film within the dose rates used. This result also indicates that the quantity of reaction products depends on the extent of the interfacial area, because the sandwiched film always shows a higher intensity ratio than the bilayer film regardless of the dose rate. It should be noted that the effect of electron irradiation during TEM observation on silicide formation can be negligible because the total dose of electrons during TEM observation (∼ 10^24^ e/m^2^) is ∼ 10^2^ times lower than that required for silicide formation (>2 × 10^26^ e/m^2^).

### Electron irradiation at 90 K

**Fig. 5.**
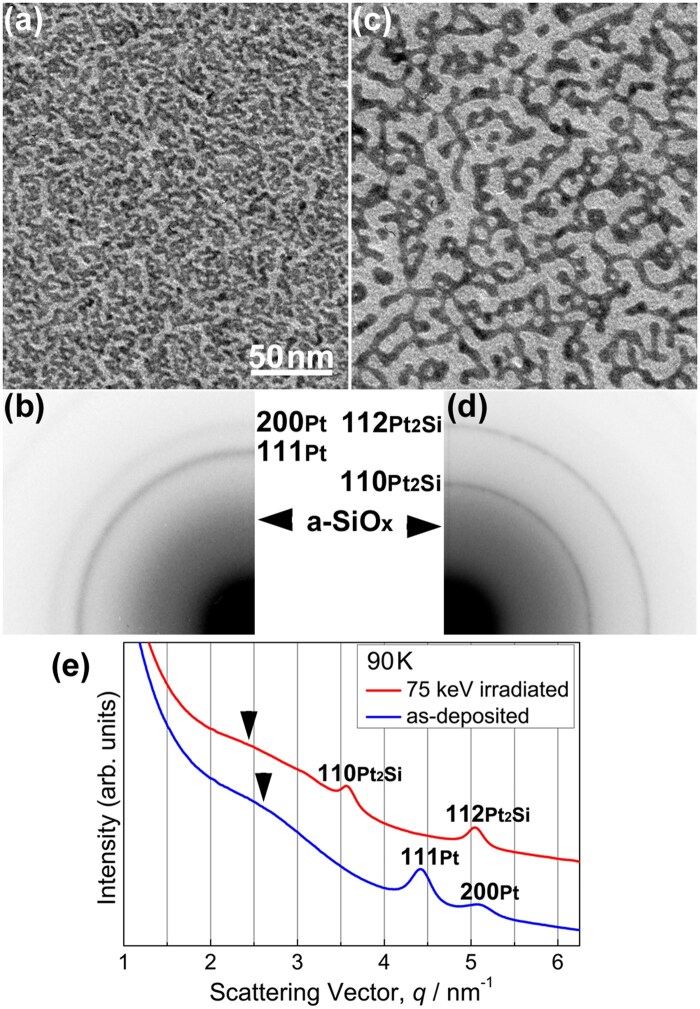
(a and b) BF-TEM images and SAED patterns, respectively, of an as-deposited a-SiO_*x*_/Pt/a-SiO_*x*_ thin film. (c and d) BF-TEM images and SAED patterns, respectively, of an a-SiO_*x*_/Pt/a-SiO_*x*_ thin film, after 75-keV electron irradiation at 90 K for 3.6 ks. The scale of (c) is the same as that of (a). (e) Intensity profiles of the SAED patterns measured in the radial direction. Reprinted with permission from Sato and Mori [21] under CC-BY-NC-ND 4.0.

Figures 5a and b show a BF-TEM image and the corresponding SAED pattern, respectively, of an as-deposited a-SiO_*x*_/Pt/a-SiO_*x*_ thin film observed at 90 K. The overall features of the microstructure were similar to those of the specimen shown in Fig. 2a and b. An interesting microstructural feature observed here is that the morphology of the metallic particles also considerably changed at 90 K after the 75 keV electron irradiation (total dose: 2.7 × 10^26^ e/m^2^) (Fig. 5c). This result indicated that the coalescence and growth of metallic particles are not due to thermal effects during electron irradiation (beam heating), but to an electronic excitation effect; i.e. we ruled out a thermal process. There are conflicting reports regarding diffusing species during Pt_2_Si formation at a Pt/Si interface. Pretorius [31] reported that Pt diffusion is dominant, whereas Poate and Tisone [32] reported that Si is the diffusing species. The dominant species are unknown in the case of Pt/a-SiO_*x*_ interfaces; however, to sustain the reaction, it is necessary to supply Si to the reaction front on the surface of the previously formed Pt–Si compound layer that exists between a metallic particle and an a-SiO_*x*_ matrix. In other words, it is conceivable that to maintain the reaction front active at the interface between metallic particles (i.e. the Pt–Si alloy or compound particles) and a-SiO_*x*_, the dissociation product (i.e. Si atom) from SiO_*x*_ should be steadily and constantly supplied to the front (in this sense, Si diffuses into the Pt-layer). One method to achieve such a supply is a drastic morphology change of the particles, which would facilitate a steady and constant supply of fresh surfaces of the Pt–Si metallic particles and consequently serve as a steady supply of new reaction sites where newly formed Si atoms may react with Pt atoms until the Pt atoms in the particles become saturated with respect to Si atoms. The considerable particle morphology change observed in Figs. 2c and 5c may correspond to such a situation and may be attributable to the need to achieve a further free energy gain of the system.

### Chemical analysis by EDS elemental mapping

**Fig. 6.**
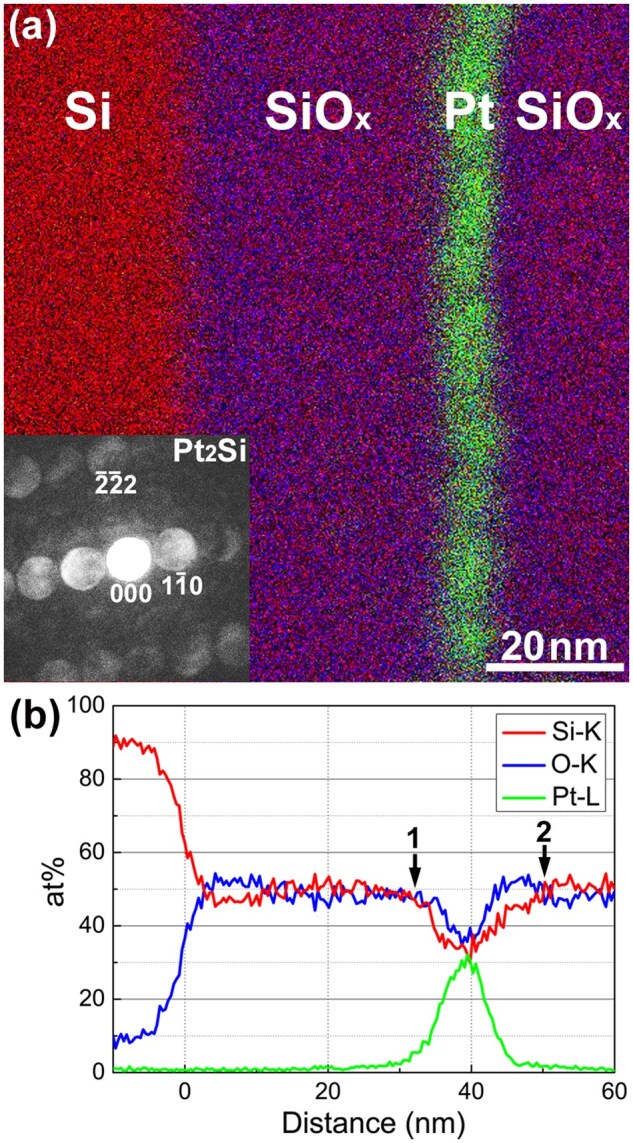
(a) STEM–EDS elemental map of a cross-section of a-SiO_*x*_/Pt/SiO_*x*_/Si(111). We irradiated the sample with 30 keV electrons at room temperature for 10.8 ks. The Pt-layer contains α-Pt_2_Si. The inset shows a nanobeam electron diffraction pattern obtained from the α-Pt_2_Si phase. (b) Composition profiles extracted from the STEM–EDS map. Arrows 1 and 2 indicate the positions where the Si concentration decreases and subsequently increases, respectively. The Si on the surface of the cross-section specimen may be covered with a native oxide. Reprinted with permission from Sato and Mori [21] under CC-BY-NC-ND 4.0.

Figure 6a shows a STEM–EDS elemental map obtained for a cross-sectional specimen fabricated from an a-SiO_*x*_/Pt/a-SiO_*x*_ thin film grown on a Si(111) substrate. Si (red), SiO_*x*_ (purple) and Pt (green) layers are evident. We irradiated the specimen with 30 keV electrons for 10.8 ks (total dose: 1.7 × 10^25^ e/m^2^) inside the dual-beam FIB before microsampling. In this case, the formation of Pt_2_Si in the Pt layer was partial, and unreacted Pt remained. This was determined from the fact that Pt reflections remained in the Debye–Scherrer rings of a plan-view specimen irradiated under the same irradiation conditions (not shown). A nanobeam electron diffraction pattern obtained from the α-Pt_2_Si phase with nearly [112] zone axis (Fig. 6a, inset) shows the electron irradiation-induced silicide formation. Pt-based metallic particles are dispersed, yet one cannot completely delineate the particles because of their overlap in the observation direction. A nanometer-scale rugged interface between Pt and SiO_*x*_ may favor appreciable atomic mixing during Pt_2_Si formation. We infer that similar microstructures also formed in the specimens shown in Figs. 2 and 5 because the sputtered Pt always forms islands on the a-SiO_*x*_ layer. Hence, it is presumed that the drastic change in morphology of the metallic particles by electron irradiation, as observed in Figs. 2c and 5c, takes place mainly in the lateral direction of the composite film, not in the film growth direction.

Figure 6b shows composition profiles extracted from the STEM–EDS map shown in Fig. 6a. We quantified the concentration (in atomic percent) based on a thin film approximation [33], assuming the theoretical k-factor (standardless quantification), and hence the derived concentrations are not quantitative. We set the total Si, O and Pt content to 100 at%. We assigned the origin of the distance to the Si/a-SiO_*x*_ interface, and the scale on the horizontal axis is the same in Fig. 6a and b. The Si concentration started to decrease at ∼ 32 nm (arrow 1), reached a minimum at 40 nm (center of the Pt location), and again increased and recovered at 50 nm (arrow 2). This change in Si concentration profile may reflect the Pt_2_Si formation at the rugged interface between Pt and SiO_*x*_ mentioned above (i.e. a-SiO_1.5_ → a-SiO_2_). The oxygen content detected at the Pt layer is due to the overlap of Pt (or Pt-Si) and SiO_*x*_ in the projection direction in the cross-­sectional TEM observation.

**Fig. 7.**
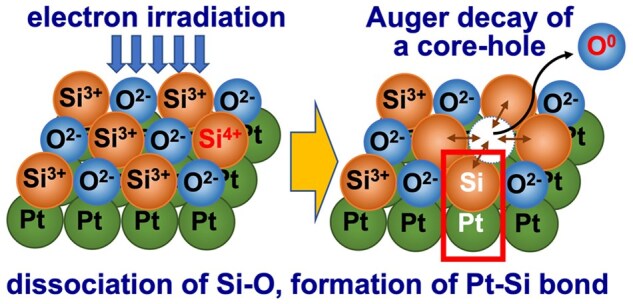
Schematic illustration showing the elementary processes of the novel interfacial reaction proposed in this study; dissociation of a Si–O bond, and formation of an Pt–Si bond. The crystal structure of α-Pt_2_Si is shown on the right. Adapted with permission from Sato and Fujii [22].

The mechanism of Pt_2_Si formation by electron irradiation is summarized below. Electronic excitation first breaks a Si–O bond, which is immediately followed by Pt–Si bond formation (or recombination of Si–O bond) at the Pt/a-SiO_*x*_ interfaces, and eventually Pt_2_Si formation leads to Si depletion in the SiO_*x*_ matrix, as described in the ‘Introduction’ section. The Si depletion was detected in an electron diffraction study; the first halo peak of amorphous SiO_*x*_ shifts to the lower scattering angle side with increasing oxygen concentration (Figs. 2e and 5e). Figure 7 shows a schematic illustration showing the elementary processes of the interfacial reaction proposed in this study: (i) electronic excitation of a-SiO_*x*_ (*x*∼1.5) (Si^3+^→Si^4+^) by electron irradiation, (ii) Auger decay of a core-hole, (iii) dissociation of a Si–O bond, and (iv) formation of a Pt-Si bond. The dissociation mechanism triggered by electronic excitation is similar to the case in the K-F model for oxygen ion desorption from the free surface of oxides [19]. Note that the illustration shown in Fig. 7 exaggerates the desorption of oxygen, as such desorption of oxygen does not occur except at the surface. Instead, recombination of Si–O bonds occurs, or it is also possible for Si atoms that become free from O atoms for a short time can be trapped by Pt atoms at the Pt/a-SiO_*x*_ interface. All these processes occur repeatedly during electron irradiation. In this manner, it is possible to form Pt_2_Si at the Pt/a-SiO_*x*_ interface.

No compounds other than α-Pt_2_Si were formed within the range of electron irradiation conditions used in this study. We can assume that Si diffuses into Pt since the dissociated Si reacts with Pt. It has been reported that the first phase formed at the Pt/Si planar interface by heat treatment is Pt_2_Si [34,35]. The heat of formation of Pt_2_Si (−47.7 kJ/mol) is larger than that of Pt_3_Si (−36.9 kJ/mol) [35]. Based on these facts, it is reasonable that Pt_2_Si is also formed at the Pt/a-SiO_*x*_ interface. Once Pt_2_Si is formed, it may be stabilized, while the reason for the stabilization is not clear. The distinctive feature of the method proposed in this study is that, unlike the Pt/Si interfacial reaction induced by heat treatment, only α-Pt_2_Si is produced.

## Fe_2_Si formation at Fe/a-SiO_*x*_ interface [22]

### A brief overview of iron silicides as functional materials

As an example of the application of the novel process to the synthesis of functional materials, we conducted research on Fe silicide formation. Fe–Si system forms various silicides that have different stoichiometric compositions, such as Fe_3_Si, Fe_2_Si, FeSi and FeSi_2_ [36]. Among these compounds, β-FeSi_2_ has attracted much interest for thermoelectric materials or light-emitting materials in the infrared region, while the formation temperature is higher than 1000 K [37–39]. On the other hand, trigonal Fe_2_Si, a ferromagnetic half-metal with a 100% spin-polarization ratio, is expected to be a novel spintronics material based on electronic structure calculations, although there are few reports on this topic [40,41]. The advantage of the Fe–Si system is that both Fe and Si are abundant resources, in addition to excellent functionality that varies depending on their chemical composition. However, synthesis of iron silicides generally requires high-temperature processes, and hence, low-temperature synthesis of nanoscale silicides via the aforementioned nonradiative transition is of technological interest. The diffusion mechanism of atoms is also an issue that requires elucidation to understand the solid-state reaction.

In this study, we aim to form nanoscale iron silicide(s) at the Fe/a-SiO_*x*_ thin film interface by electron irradiation and discuss the types of compounds formed by electronic excitation.

### Electron irradiation at 298 K

**Fig. 8.**
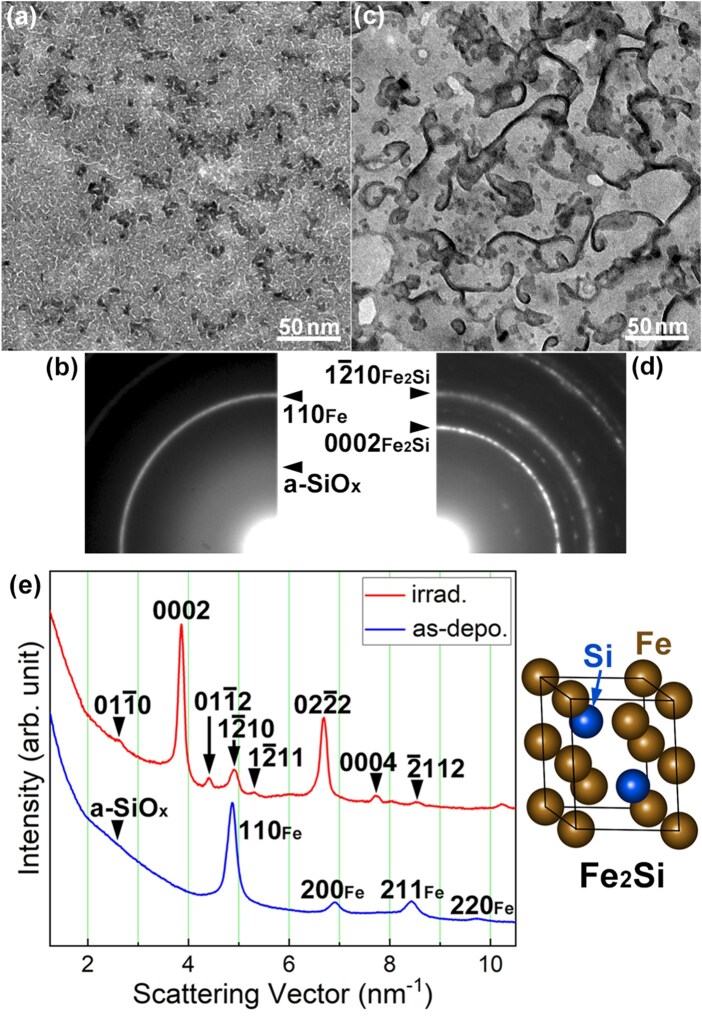
BF-TEM images and SAED patterns of an a-SiO_*x*_/Fe/a-SiO_*x*_ thin film. (a and b) as-deposited, (c and d) after 75-keV electron irradiation at 298 K for 7.2 ks (total dose: 1.0 × 10^28^ e/m^2^). (e) Intensity profiles of the SAED patterns obtained for the as-deposited and the irradiated specimens. The crystal structure of Fe_2_Si is shown in the inset. Adapted with permission from Sato and Fujii [22].

Figures 8a and b show a BF-TEM image and the corresponding SAED pattern of an as-deposited a-SiO_*x*_/Fe/a-SiO_*x*_ thin film, respectively. A maze-like contrast is seen on the BF-TEM image. The SAED pattern is composed of Debye–Scherrer rings of body-centered cubic (bcc) Fe and a weak halo ring of a-SiO_*x*_. Thus, a maze-like contrast seen in the BF-TEM image arose from the discontinuous bcc-Fe thin film. No diffraction spots originating from iron oxide were observed. After 75 keV electron irradiation at 298 K for 7.2 ks, the morphology of the thin film significantly changed, as shown in Fig. 8c. The electron dose rate was 1.4 × 10^24^ e/m^2^s (total dose: 1.0 × 10^28^ e/m^2^). Extensive coalescence and growth of Fe nanostructures occurred during electron irradiation. Fig. 8d shows the SAED pattern after 75 keV electron irradiation. As can be seen, Debye–Scherrer rings other than bcc-Fe appeared after electron irradiation. These diffraction rings can be indexed by the trigonal Fe_2_Si phase (Fe_2_Si-type structure, *P*$\overset{-}{3}$*m*1) [42]. The crystal structure is shown in the inset. It should be noted that under 75 keV electron irradiation, atomic migration induced by knock-on atom displacement can be excluded since the electron energy of 75 keV is below the threshold of knock-on atom displacement both for Fe (370 kV) [43] and Si (216 kV or 197 kV for Si in SiO_2_) [30]. Therefore, the formation of Fe_2_Si was likely caused by the same mechanism as in the Pt/a-SiO_*x*_ system, i.e. the dissociation of a-SiO_*x*_ due to electronic excitation [17]. That is, Si–O bonds dissociate through Auger decay of core-holes generated by electronic excitation, and then Fe–Si bonds are formed. Figure 8e shows the intensity profiles of the SAED patterns obtained for the as-deposited and the irradiated specimens. The intensity was integrated in the circumferential direction. The as-deposited specimen was composed of bcc-Fe and a-SiO_*x*_. Intensity profiles clearly show the formation of Fe_2_Si upon electron irradiation. Careful inspection of the diffraction pattern revealed the existence of a small amount of unreacted bcc-Fe. Fe_2_Si was also formed by 75 keV electron irradiation at 90 K, while the reaction was somewhat reduced with no significant grain growth [22].

**Fig. 9.**
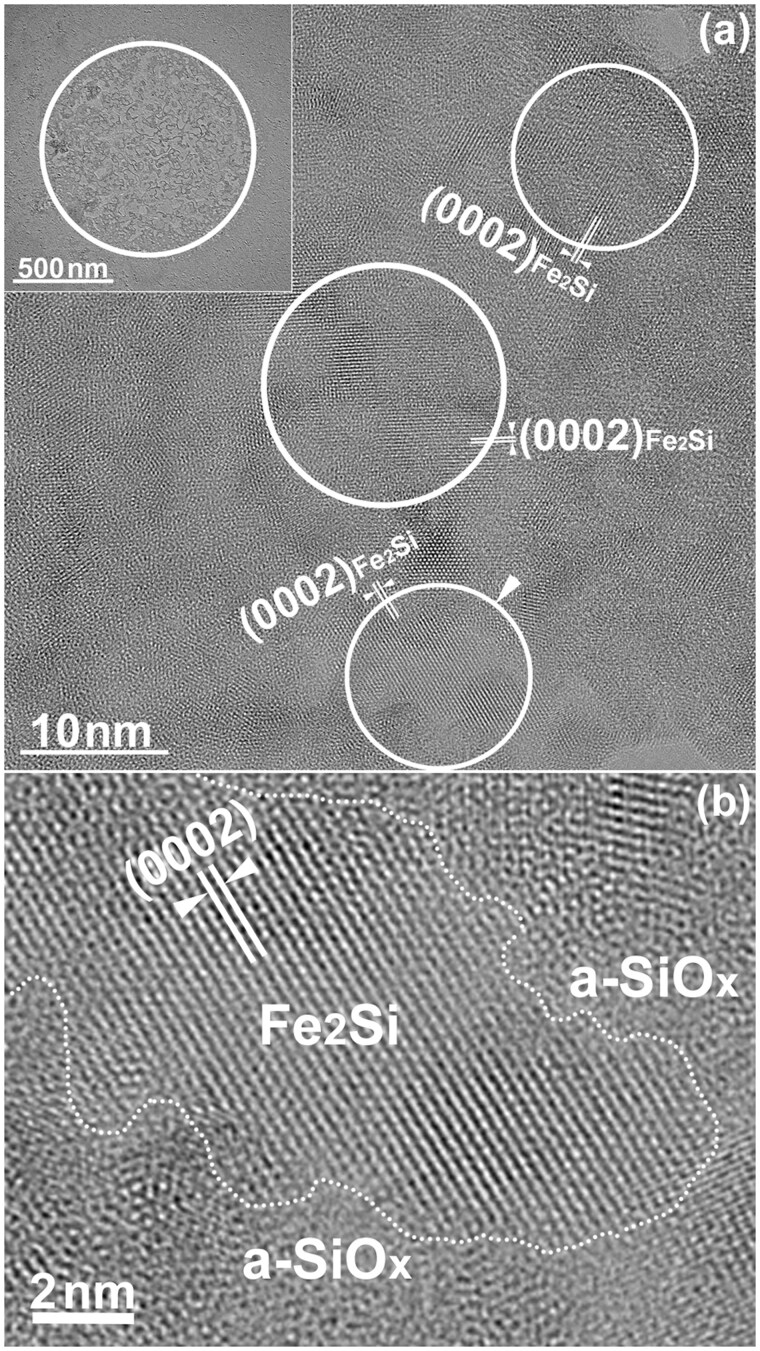
(a) HRTEM image observed in the irradiated area of the specimen shown in Fig. 8c. The upper left inset indicates the area where significant morphological change occurred. (b) A magnified HRTEM image of the area indicated by an arrowhead in (a), including the interface between Fe_2_Si and a-SiO_*x*_. Reprinted with permission from Sato and Fujii [22].

Figure 9a shows an HRTEM image observed in the irradiated area of the specimen shown in Fig. 8c. The upper left inset shows the entire area where significant coalescence and growth occurred by electron irradiation. The HRTEM image shows distribution of (0002) lattice fringes of the trigonal Fe_2_Si with lattice spacing of 0.25 nm. The size of the region containing Fe_2_Si is ∼ 10 nm in diameter, and each grain oriented in different directions. Thus, formation of the Fe_2_Si in a local region was confirmed. Figure 9b shows a magnified HRTEM image of the area indicated by an arrowhead in Fig. 9a, including the interface between Fe_2_Si and a-SiO_*x*_. Clear (0002) lattice fringes are observed in the Fe_2_Si region. The interface between the crystalline and amorphous phases is indicated by a dotted line. As seen, the interface is not flat, but has a complex shape. No compounds such as Fe oxides other than Fe_2_Si or a-SiO_*x*_ are observed at the interface.

As mentioned above, Fe_2_Si was formed by 75 keV electron irradiation on the Fe/a-SiO_*x*_ thin film. The result shows the robustness of the methodology; a silicide formation is possible for elements that have a lower affinity for oxygen than Si. Walser and Bené [34] proposed a rule for the first compound formed at the planar interface between transition metal and Si. According to their study, equiatomic FeSi was the predicted compound and was, in fact, observed between Fe and Si. Similarly, the effective heat of formation model proposed by Pretorius *et al*. [35] predict the formation of FeSi as the first phase formed between Fe and Si. The results of this study appear to contradict these thermodynamical predictions, but this may be due in part to the difference in interface morphology, i.e. compound formation at planar interfaces by thermal annealing versus that at complicated nanometer-scale interfaces by electronic excitation. In the present study, it is atomic Si rather than planar Si that forms the interface with Fe, and the above model may not be directly applicable. As discussed for the Pt/a-SiO_*x*_ system (see description of Fig. 7), it is considered that the dissociated Si diffuses into the metal layer, which may also be the reason that the Fe-rich compound is formed at the beginning of the reaction instead of equiatomic FeSi.

**Fig. 10.**
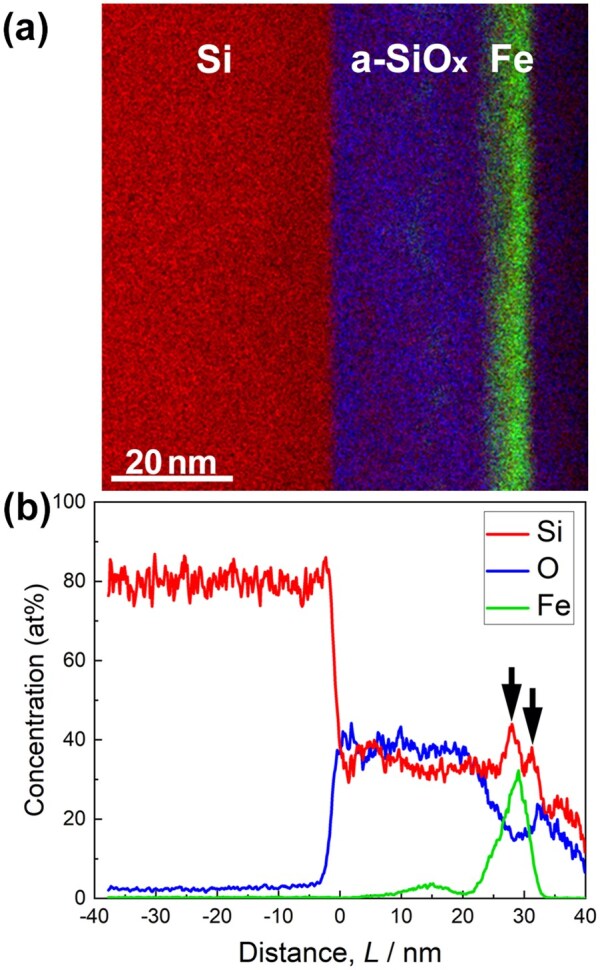
(a) STEM-EDS elemental map of the cross-section of a-SiO_*x*_/Fe/a-SiO_*x*_/Si(111) after 75 keV electron irradiation at 298 K for 7.2 ks (total dose: 1.0 × 10^28^ e/m^2^). The Fe-layer contains Fe_2_Si. (b) Composition profiles extracted from the STEM-EDS map. Reprinted with permission from Sato and Fujii [22].

Figure 10a shows the STEM-EDS elemental map obtained for a cross-sectional specimen after 75 keV electron irradiation at 298 K. Distribution of Fe (green), Si (red) and O (blue) are evident. The total electron dose irradiated was 1.0 × 10^28^ e/m^2^ at 75 keV. As the elemental map shows, the Fe-rich layer maintains its layered structure even after electron irradiation. This tendency is consistent with the results for the Pt/a-SiO_*x*_ system (Fig. 6a). Figure 10b shows concentration profiles extracted from the STEM-EDS elemental map shown in Fig. 10a, derived based on the thin film approximation [33], assuming the theoretical k-factor. A noteworthy point is that the Si concentration increases significantly at the Fe/a-SiO_*x*_ interfaces, as indicated by arrows. The local increase in Si concentration in electron-irradiated a-SiO_*x*_ is direct evidence for the supply of Si via the dissociation of a-SiO_*x*_. The increase in Si concentration is remarkable compared with the results for the Pt/a-SiO_*x*_ system. This is because the electron flux used in this study was 20 times higher than that used in the Pt/a-SiO_*x*_ case, and hence, the dissociation of a-SiO_*x*_ could have been enhanced. However, it should be noted that differences in the surface coverage of the metal or metal silicide layer also affect the shape of the Si concentration profile in cross-sectional observation, making quantitative analysis difficult. Also note that a small bump in Fe concentration near the distance of 15 nm is presumed to be an artifact due to redeposition during FIB microsampling.

**Fig. 11.**
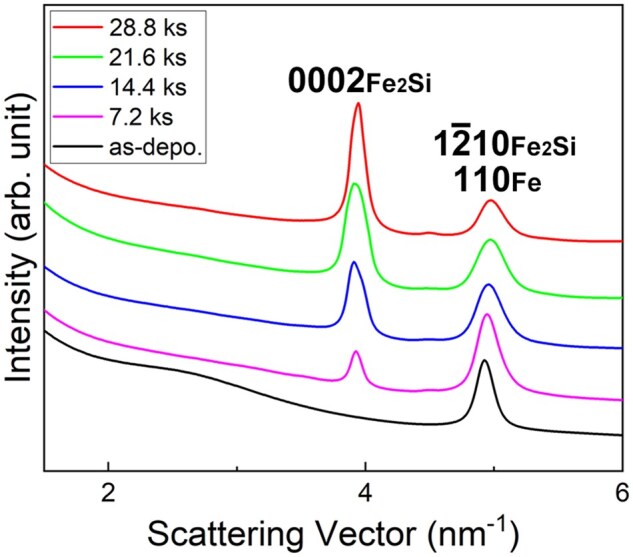
Intensity profiles of the SAED patterns obtained for the specimens with different irradiation times. The total doses are as follows: 1.0 × 10^28^ e/m^2^ (7.2 ks), 2.0 × 10^28^ e/m^2^ (14.4 ks), 3.0 × 10^28^ e/m^2^ (21.6 ks), 4.0 × 10^28^ e/m^2^ (28.8 ks). Reprinted from Supporting Information of Sato and Fujii [22].

To explore the possibility of the formation of other types of compounds besides Fe_2_Si, we attempted long-term electron irradiation up to 28.8 ks (total dose: 4.0 × 10^28^ e/m^2^), but no Fe-Si compounds other than Fe_2_Si were formed. Figure 11 shows the intensity profiles of the SAED patterns obtained for the specimens with different irradiation times. As the electron irradiation proceeded, intensity of 0002_Fe2Si_ reflection increased, and the peak position of 110_Fe_ and/or 1$\overset{-}{2}$10_Fe2Si_ reflection shifted slightly toward higher scattering angles. Since the 110_Fe_ reflection (4.934 nm^−1^) almost overlaps with the 1$\overset{-}{2}$10_Fe2Si_ (4.936 nm^−1^), the above-mentioned change indicates Fe consumption associated with the Fe_2_Si formation. Under the irradiation conditions used in this study, no signs of the formation of Fe–Si compounds other than Fe_2_Si were observed. This means that Si concentration did not increase more than ∼ 33at% (Fe_2_Si) during the prolonged electron irradiation up to 28.8 ks. The factors that stabilize Fe_2_Si remain unresolved. It should be mentioned that Fe_2_Si formation was slight at a total dose of 5 × 10^27^ e/m^2^ regardless of the dose rate (3.8 × 10^23^–1.4 × 10^24^ e/m^2^s). This is significantly different from the case of Pt/a-SiO_*x*_ system, in which Pt_2_Si was formed at a dose of 2.7 × 10^26^ e/m^2^ [21]. This is presumably due to the difference in heat of formation between Pt_2_Si (−47.7 kJ/mol [35]) and Fe_2_Si (−23 kJ/mol [44]).

**Fig. 12.**
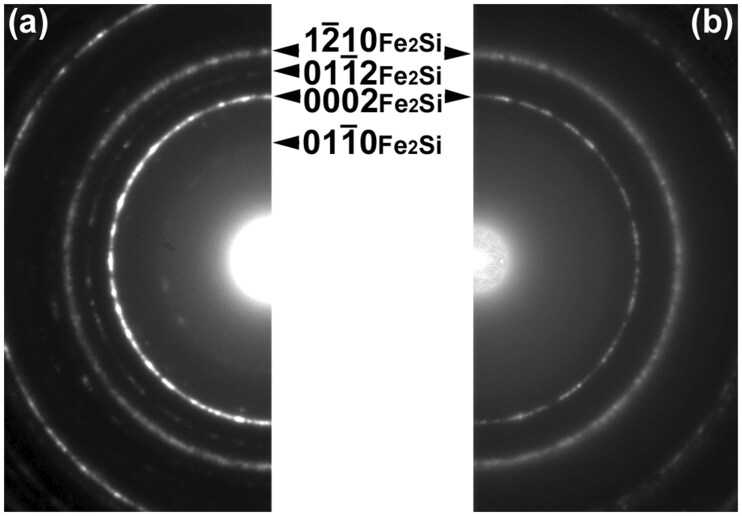
SAED patterns obtained for a-SiO_*x*_/Fe/a-SiO_*x*_ (a) and a-SiO_2_/Fe/a-SiO_2_ (b) thin films after 75 keV electron irradiation at 298 K (total dose: 1.0 × 10^28^ e/m^2^). Reprinted from Supporting Information of Sato and Fujii [22].

We also examined the effect of oxygen content on the silicide formation; namely, a similar irradiation experiments were conducted using a-SiO_2_ instead of a-SiO_*x*_ (*x* ∼ 1.5). a-SiO_2_/Fe/a-SiO_2_ thin films were fabricated by radio-frequency (RF) magnetron sputtering on NaCl(001) substrates. Figure 12 compares the SAED patterns obtained for a-SiO_*x*_/Fe/a-SiO_*x*_ (Fig. 12a) and a-SiO_2_/Fe/a-SiO_2_ (Fig. 12b) thin films after 75 keV electron irradiation at 298 K (total dose: 1.0 × 10^28^ e/m^2^). As seen, Fe_2_Si was also formed in the a-SiO_2_/Fe/a-SiO_2_ thin film, while some of the reflections such as 01$\overset{-}{1}$0 and 01$\overset{-}{1}$2 are not visible. This indicates that Fe_2_Si formation is slower at the Fe/a-SiO_2_ interface than that at the Fe/a-SiO_*x*_. This is presumably because a-SiO_2_ is chemically more stable than a-SiO_*x*_ (*x* ∼ 1.5), and Si-O bonds are less likely to dissociate due to electronic excitation. Chen *et al*. [45] reported the formation of Si nanostructures in a-SiO_2_ thin film by 100 keV-electron irradiation with a high electron dose (3 × 10^28^ e/m^2^) similar to the present study (1–4 × 10^28^ e/m^2^), while they used electron flux of ∼ 7.5 × 10^27^ e/m^2^s, which was three orders of magnitude higher than that used in this study (1.4 × 10^24^ e/m^2^s). They stated that a-SiO_2_ is very insensitive to electron-beam irradiation, requiring a threshold dose as high as 10^28^ e/m^2^ to transform completely into silicon. Thus, based on previous findings and the results obtained in this study, it is evident that a-SiO_*x*_ is more easily dissociated by electron irradiation compared to a-SiO_2_. In another study, Du *et al*. [46] reported that crystalline Si nanoparticles were formed in a-SiO_2_ by 200 keV electron irradiation for several minutes at a dose rate of 10^24^ e/m^2^s. They attributed the origin of the structural change to the reduction of a-SiO_2_ by valence electron excitation, followed by the crystallization of a-Si by beam heating and knock-on atom displacement. The mechanism of the above reaction appears to be different from that of our study.

Interfacial solid-state reaction similar to that in this study has been reported for Pd/Al_2_O_3_ system; reduction of Al_2_O_3_ was induced by 300 keV electron irradiation, which eventually led to the formation of metastable Al_2_Pd [47]. In the Pd/SiO_*x*_ system, an amorphous Pd–Si phase was formed by irradiation with 25–200 keV electrons [48]. Thus, dissociation of oxides by electronic excitation is a highly versatile method for producing metal–metalloid or intermetallic compounds at room temperature. Electronic excitation also induces crystallization of amorphous Al_2_O_3_ [49], Ge [50,51] and SiGe thin films [52,53]. In particular, references [51] and [53] report the existence of a minimum threshold of electron flux for crystallization by low-energy electron irradiation (2–20 keV). These reports demonstrate that electronic excitation is certainly useful for the creation and modification of novel materials. In the next section, the effect of electronic excitation on the crystallization of an amorphous alloy thin film will be demonstrated, taking the Pd–Si system as an example.

## Solute-atom-mediated crystallization of an amorphous alloy thin film [23]

### Athermal crystallization induced by electron irradiation

In general, amorphous materials are thermodynamically metastable, and they transform to equilibrium phase(s), i.e. crystalline solids, with the assistance of thermal energy [54]. It is known in most cases that crystallization proceeds toward stable phase(s) via several metastable phases [55]. Also, it is known that, the thermal stability of the amorphous phase was enhanced by increasing the number of constituent components, which contributed to the development of bulk metallic glasses [56]. If we confine ourselves to inorganic amorphous materials, the origin of crystallization is not limited to thermal energy, but an ionization process can also induce crystallization. Such an athermal crystallization has been reported for oxide compounds [57], Al_2_O_3_ [49], Si [58], Ge [50,51,58] and SiGe [52,53]. In these studies, the origin of the crystallization has been attributed to electronic excitation since the crystallization proceeds under irradiation conditions where knock-on atom displacement is absent. However, it is critical to discriminate whether the origin of the crystallization is purely ionization or includes thermal energy assistance as well.

The Pd–Si alloy system is a famous amorphous forming system by quenching from the melt [59]. In a normal heat treatment, Pd_3_Si is formed via precipitation of several metastable phases [60]. On the other hand, Nagase *et al*. [61] reported that Pd_2_Si was formed by electron irradiation at room temperature, mainly based on the experimental results of 200 keV electron irradiation at 298 K. The important point here is that the amorphous Pd–Si (hereafter, a-Pd–Si) was in contact with a-SiO_*x*_ (*x* ∼ 1.5) in their work. A possible role of a-SiO_*x*_ upon crystallization is considered to be the dissociation of the oxide induced by electronic excitation. Regarding this point, we have studied the dissociation of a-SiO_*x*_ using photon irradiation and found that excitation of Si2p electrons, followed by Auger decay of the core-hole is responsible for the dissociation process [17,18]. The dominant valence state was Si^3+^ before photon irradiation, and then, Si^4+^ and Si^0^ markedly increased after photon irradiation [18]. Thus, it is presumed that the crystallization of a-Pd–Si could be directly related to the dissociation of a-SiO_*x*_ by electronic excitation.

In this study, we have made clear the effect of additional Si on the electron irradiation-induced crystallization of a-Pd–Si alloy thin films using TEM and electron diffraction. Based on the results, a new mechanism of crystallization of amorphous alloys mediated by additional solute atoms is proposed.

### Electron irradiation of a freestanding a-Pd–Si alloy thin film

**Fig. 13.**
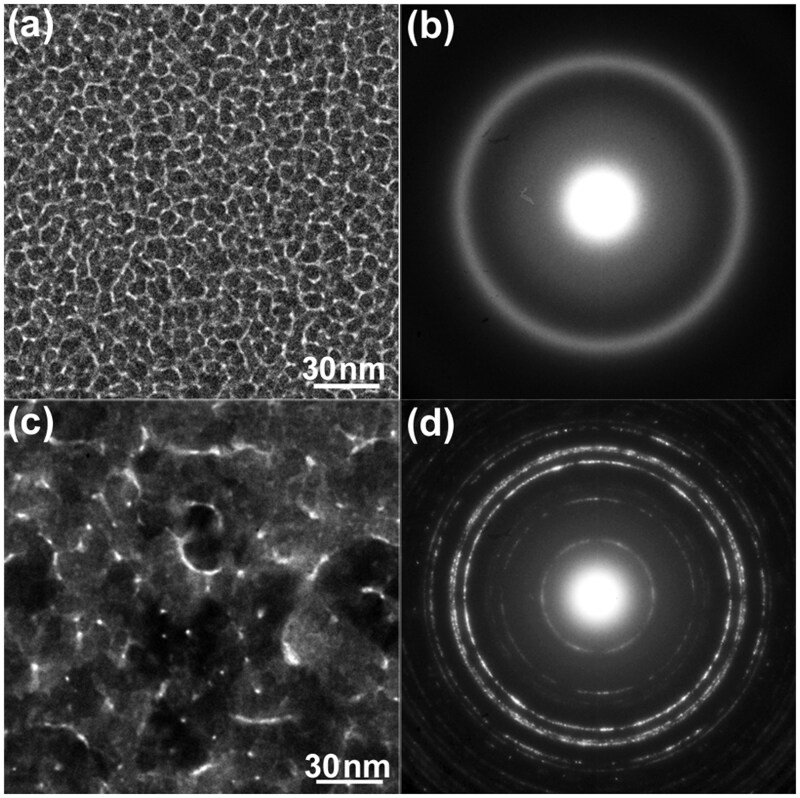
BF-TEM images and corresponding SAED patterns of an a-SiO_*x*_/a-Pd–Si/a-SiO_*x*_ composite thin film. (a and b) as-deposited, (c and d) after 75 keV-electron irradiation at 298 K for 900 s (total dose: 2.7 × 10^27^ e/m^2^). Reprinted with permission from Sato and Mori [23] under CC-BY 4.0.

**Fig. 14.**
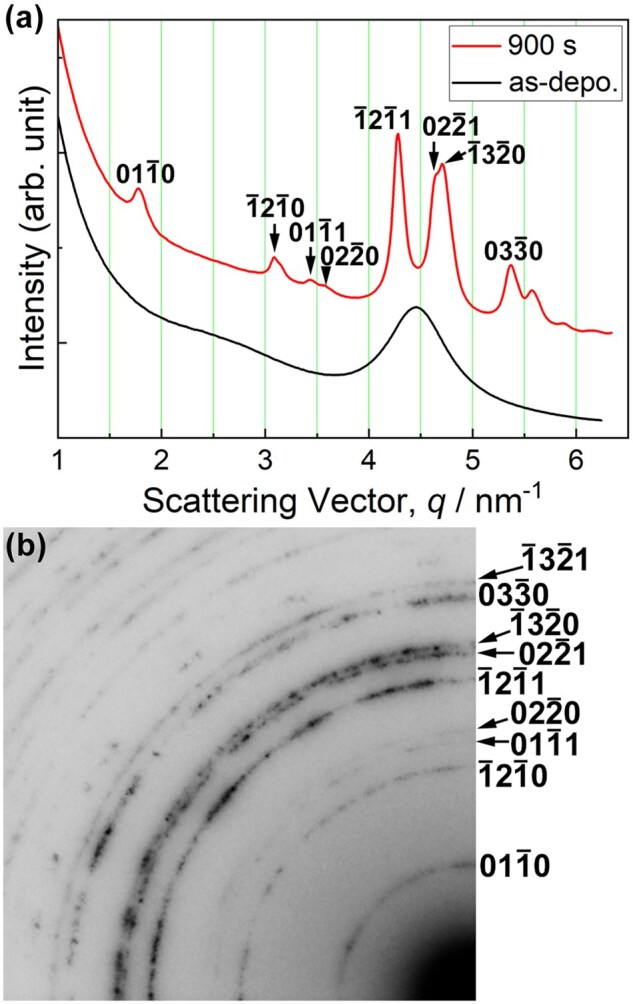
(a) Electron diffraction intensity profiles of the as-deposited and the 75 keV electron irradiated a-SiO_*x*_/a-Pd–Si/a-SiO_*x*_ composite thin film. (b) A part of the SAED pattern after electron irradiation at 298 K for 900 s. Reprinted with permission from Sato and Mori [23] under CC BY-4.0.

**Fig. 15.**
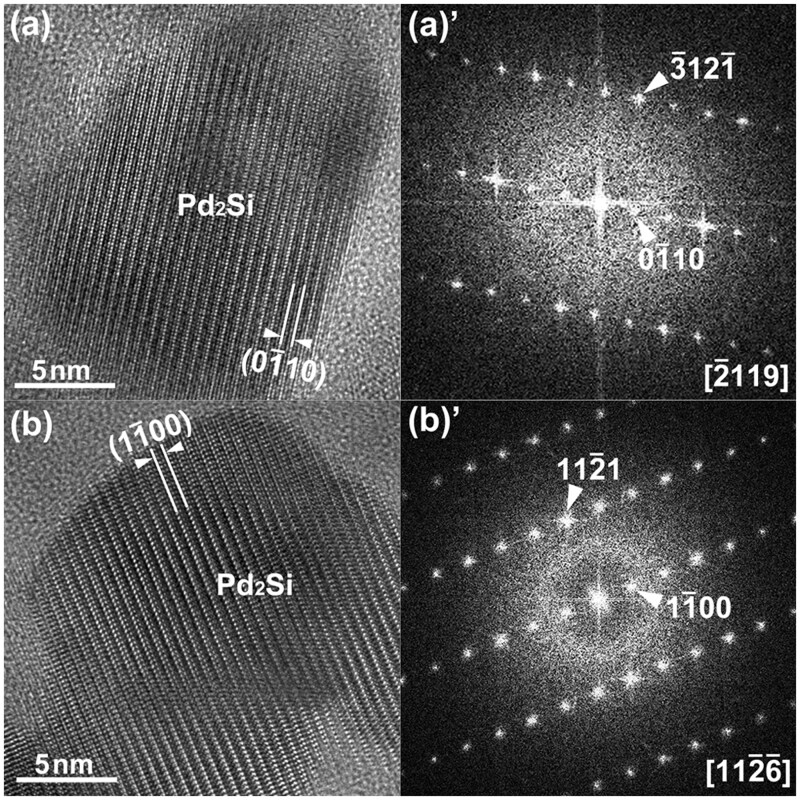
HRTEM images and FFT patterns of the tetragonal Pd_2_Si formed by 75 keV electron irradiation at 298 K (total dose: 2.5 × 10^27^ e/m^2^). The beam incidence directions for Pd_2_Si are (a)(a)’ [$\overset{-}{2}$119] and (b)(b)’ [11$\overset{-}{2}\overset{-}{6}$].

**Fig. 16.**
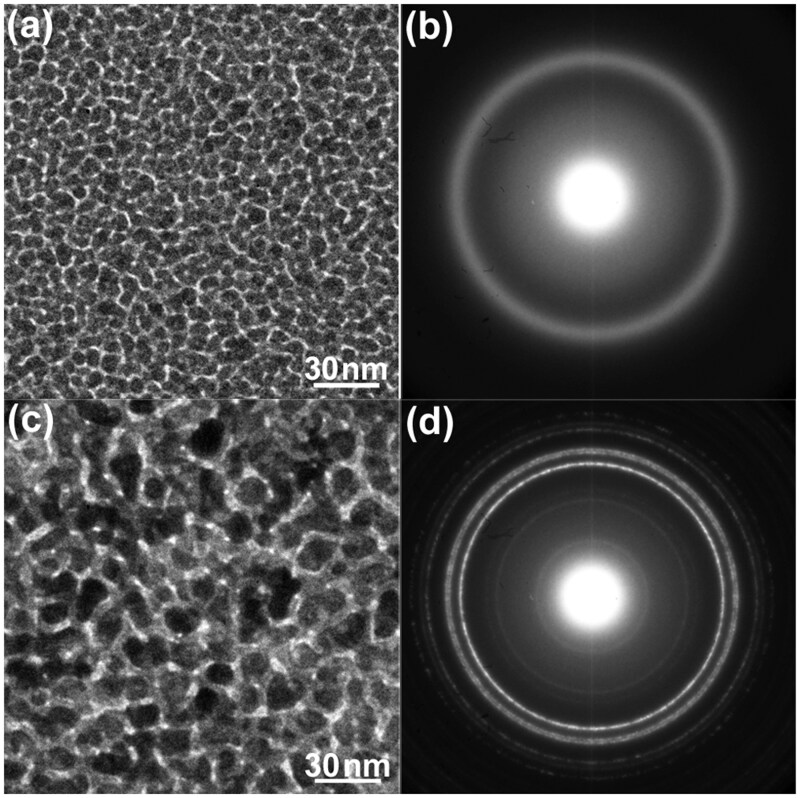
BF-TEM images and corresponding SAED patterns of an a-SiO_*x*_/a-Pd–Si/a-SiO_*x*_ composite thin film observed at 90 K. (a and b) as-deposited, (c and d) after 75 keV electron irradiation for 900 s (total dose: 2.7 × 10^27^ e/m^2^). Reprinted with permission from Sato and Mori [23] under CC-BY 4.0.

We have examined electron irradiation experiments on a freestanding a-Pd-19at%Si alloy film with 75 keV electrons at 298 K (dose rate: 1.0 × 10^24^ e/m^2^s). We used a relatively thick film (∼ 100 nm) to reduce the effect of possible surface oxidation of the freestanding film. After the electron irradiation for 2.9 ks (total dose: 2.9 × 10^27^ e/m^2^), a halo pattern of a-Pd–Si changed to diffuse Debye–Scherrer rings [23]. This result indicates an early stage of crystallization induced by electron irradiation. Actually, in the initial stage of thermal crystallization of the a-Pd_80_Si_20_ alloy, the appearance of a number of small Pd crystallites with an fcc structure was reported in the literature [60]. However, it should be emphasized here that the change observed in the SAED pattern is completely different from those observed in the a-SiO_*x*_/a-Pd–Si/a-SiO_*x*_ composite films shown later in Figs. 13–16. Also note that under 75 keV electron irradiation, knock-on atom displacement is excluded both for Pd and Si [30,43]. Similar results were also obtained with 200 keV electron irradiation [23]. Since a-Pd–Si is a metal with a low density of states at the Fermi level [62], it is presumed that the effect of electronic excitation on structural changes is weak due to charge screening.

### Electron irradiation of a-SiO_*x*_/a-Pd–Si/a-SiO_*x*_ composite thin films

Figure 13a and b show a BF-TEM image and the corresponding SAED pattern of an as-deposited a-SiO_*x*_/a-Pd–Si/a-SiO_*x*_ composite thin film, respectively. The total thickness of the composite film is ∼ 40 nm. Granular microstructure with a rather uniform contrast is seen in the BF-TEM image. A halo pattern indicates the formation of amorphous phase of Pd–Si as well as the presence of a-SiO_*x*_. After 75 keV electron irradiation at 298 K for 900 s, extensive coalescence and growth of the microstructure occurred, as shown in Fig. 13c. The electron dose rate was 3.0 × 10^24^ e/m^2^s (total dose: 2.7 × 10^27^ e/m^2^). A halo pattern of the as-deposited specimen changed to sharp but discontinuous Debye–Scherrer rings (Fig. 13d). The crystallized phase was judged to be hexagonal Pd_2_Si (Fe_2_P-type structure, *P*$\overset{-}{6}$2*m*) [42,63] based on the analysis of SAED patterns (detailed indexing is shown in Fig. 14; the crystal structure is shown in the inset of Fig. 18b). This phase is identical to that reported in the preceding study on a-(Pd–Si)/SiO_*x*_ [61], but differs from the orthorhombic Pd_3_Si obtained by annealing Pd_80_Si_20_ amorphous alloy ribbons [60]. It is emphasized here that the crystallization behavior of the composite film essentially differs from that observed in the freestanding a-Pd–Si film. Namely, a notable feature is that the crystallization process is completely different between the composite film and the free-standing film, both in terms of crystallization rate and the resulting crystalline phase. The effect of a-SiO_*x*_ films on the crystallization of a-Pd–Si will be discussed later.

Figure 14a shows electron diffraction intensity profiles of the as-deposited and the 75 keV electron-irradiated specimens. The intensities were integrated in the circumference direction in each diffraction pattern. After electron irradiation, crystallization can be recognized by the appearance of sharp diffraction peaks such as $\overset{-}{1}$2$\overset{-}{1}$1 and $\overset{-}{1}$3$\overset{-}{2}$0. A part of the SAED pattern after irradiation for 900 s is shown in Fig. 14b with Miller indices. In this pattern, the absence of a halo ring, for example, one near the 02$\overset{-}{2}$1 reflection, indicates that a rather complete crystallization was induced by the irradiation.

Figure 15 shows HRTEM images of the hexagonal Pd_2_Si formed by 75 keV electron irradiation at 298 K. The total dose irradiated was 2.5 × 10^27^ e/m^2^. FFT patterns of each image are also shown in the right panels. By analyzing the crossed lattice fringes and their lattice spacings, the beam incidence directions for crystalline Pd_2_Si were determined as follows: (a)(a)’ [$\overset{-}{2}$119] and (b)(b)’ [11$\overset{-}{2}\overset{-}{6}$]. The lattice spacing of the {0$\overset{-}{1}$10} of the hexagonal Pd_2_Si is 0.56 nm.

Figure 16a and b shows a BF-TEM image and the corresponding SAED pattern of an as-deposited a-SiO_*x*_/a-Pd–Si/a–SiO_*x*_ composite thin film observed at 90 K, respectively. Fig. 16c and d shows those after 75 keV electron irradiation at 90 K for 900 s, respectively. The electron dose rate was 3.0 × 10^24^ e/m^2^s (total dose: 2.7 × 10^27^ e/m^2^). Certainly, coalescence and growth of the microstructure occurred also at this reduced temperature, as shown in Fig. 16c, and Debye–Scherrer rings indicate formation of the hexagonal Pd_2_Si phase (Fig. 16d). Although the irradiation conditions except temperature were the same as that employed at 298 K in the experiments shown in Fig. 13, the granular microstructure remains, albeit the grain growth, and hence the Debye–Scherrer rings are continuous. This is in sharp contrast to the very rapid grain growth at 298 K (Fig. 13c), where Debye–Scherrer rings became discontinuous (Fig. 13d). This fact that grain growth at 298 K is very rapid as compared with that at 90 K may suggest that the migration rate of Pd and Si for the Pd_2_Si compound formation depends on temperature in a natural manner. Anyway, it can be concluded that crystallization by electronic excitation is realized by 75 keV irradiation at 90 K at a somewhat reduced rate as compared to the irradiation at 298 K.

It is noted that the hexagonal Pd_2_Si phase was also obtained by 200 keV electron irradiation in the a-SiO_*x*_/a-Pd–Si/a-SiO_*x*_ composite thin films, both at 298 K and 100 K [23]. The overall features obtained are essentially the same as those obtained for 75 keV electron irradiation described above. Microstructural coalescence and growth during crystallization were more pronounced with 75 keV irradiation than with 200 keV irradiation. This is because as the electron energy decreases, the ionization cross section of the core electron increases [20]. Under 200 keV electron irradiation, knock-on atom displacement of Pd is excluded, and that of Si may be insignificant (threshold: 197 kV) [30].

**Fig. 17.**
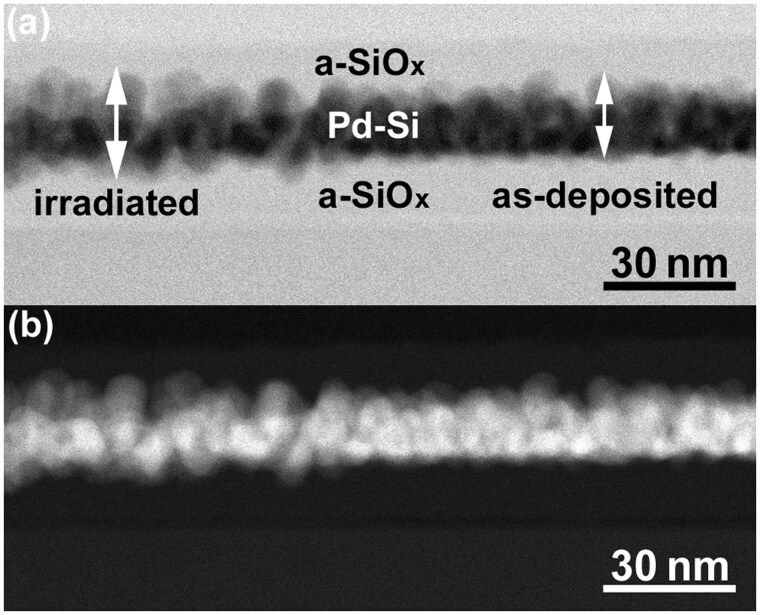
Cross-sectional STEM images observed at the interface between the irradiated and as-deposited area. (a) BF-STEM image and (b) HAADF-STEM image. Reprinted with permission from Sato and Mori [23] under CC-BY 4.0.

Figure 17a shows a cross-sectional BF-STEM image observed near the interface between the irradiated (left side of the image) and as-deposited area (right side of the image) after 200 keV electron irradiation at 298 K (total dose: 5.0 × 10^26^ e/m^2^). Pd–Si layer is imaged as a dark contrast sandwiched by a-SiO_*x*_ layers with gray contrast. As seen, it is obvious that the Pd–Si layer thickness of the irradiated region is thicker by ∼ 3 nm than that of the non-irradiated region. Figure 17b shows a High-Angle Annular Dark-Field (HAADF)-STEM image obtained from the same area shown in Fig. 17a. The spread of the Pd–Si layer is clearly observed with bright contrast (atomic number contrast). According to the EDS analysis, the chemical composition of the central part of the irradiated Pd–Si layer was Pd-31at%Si, which is close to Pd_2_Si. Note that Si concentration increased due to the compound formation (initial alloy composition was Pd-19at%Si).

### Crystallization mechanism under electron irradiation

Dissociation of a-SiO_*x*_ occurs via electronic excitation induced by electron irradiation (75 and 200 keV). This process involves core electron excitation; Auger decay of the core-hole is responsible for the dissociation of a-SiO_*x*_ [17,18]. In our previous photon irradiation experiments (photon energy of 80–680 eV), we found that the excitation of Si2p electrons (binding energy of 99 eV) is essential for the dissociation of a-SiO_*x*_ [17]. In contrast, it should be noted that 75 keV electrons can excite all core electrons, including the ground state. One of the dissociation products (i.e. free Si) is highly reactive and hence it immediately forms a chemical bond with an adjacent atom or simply returns to the original state (a-SiO_*x*_). Dissociation of a-SiO_*x*_ by electronic excitation contributes essentially to the crystallization of a-Pd–Si in the composite film, since the structural change of the freestanding a-Pd–Si film due to a similar electron irradiation is remarkably small in degree and different in nature. If dissociated Si atoms dissolve into the a-Pd–Si layer across the a-Pd–Si/a-SiO_*x*_ interface, then the composition of the a-Pd–Si layer (initially Pd-19at%Si) shifts toward higher Si content. In fact, a composition of Pd-31at%Si was obtained in the central part of the Pd–Si layer after electron irradiation. In our previous study on Pt/a-SiO_*x*_ system [17], it was found that composition of the a-SiO_*x*_ (*x*∼ 1.5) shifts toward stable SiO_2_ with the formation of Pt_2_Si (this can be confirmed by the shift of the first halo ring position of a-SiO_*x*_). It should be mentioned that the amount of Si atoms necessary for the Pd_2_Si formation from Pd-19at%Si is one-third of those required for the Pt_2_Si formation from pure Pt. The a-Pd–Si layer is sandwiched between a-SiO_*x*_ layers, and when a part of the a-SiO_*x*_ is dissociated by electronic excitation, then chemically active Si atoms can be alloyed with the a-Pd–Si layer across the interface.

**Fig. 18.**
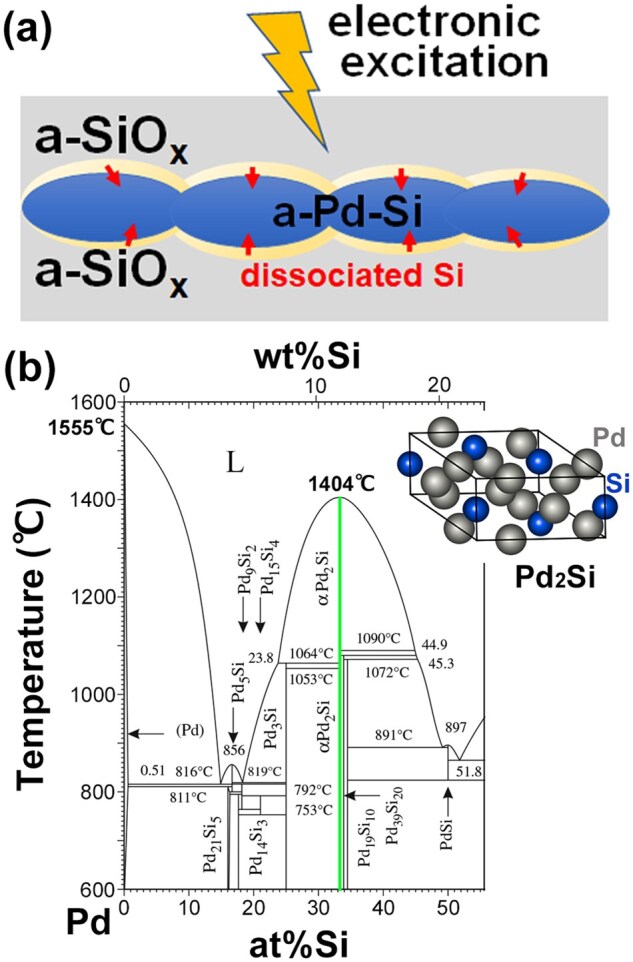
(a) Schematic illustration of dissolution of naked Si atoms into a-Pd–Si layer induced by dissociation of a-SiO_*x*_ under electron irradiation. Electronic excitation first breaks a Si–O bond, which is immediately followed by alloying of the dissociated Si with a-Pd–Si at the interfaces (pale yellow region), and eventually, leads to the Pd_2_Si formation. (b) Equilibrium phase diagram of the Pd–Si system [64]. The crystal structure of Pd_2_Si is shown in the inset. Reprinted with permission from Sato and Mori [23] under CC-BY 4.0.

Figure 18a shows a schematic of Pd_2_Si formation at the a-Pd–Si/a-SiO_*x*_ interface by electron irradiation. Electronic excitation first breaks a Si−O bond, which is immediately followed by alloying of dissociated Si with a-Pd–Si at the interfaces and eventually leads to the crystalline Pd_2_Si formation. However, energy transfer due to nonradiative relaxation after electronic excitation would not be enough for atoms to migrate over a long distance. To sustain the crystallization, it is necessary to supply Si to the reaction front on the surface of the previously formed Pd_2_Si crystallite that exists between an amorphous Pd–Si and an a-SiO_*x*_ layer. One method of achieving such a supply is an extensive morphology change of the grains, which would facilitate a steady and constant supply of active reaction front. Morphology change observed in Figs. 13c (298 K) and 16c (90 K) may correspond to such a situation. A prominent morphology change was also observed even at 90 K in the case of α-Pt_2_Si formation at Pt/a-SiO_*x*_ interface (Fig. 5c).

Based on the above considerations, it is reasonable to interpret that the observed slight increase of the a-Pd–Si layer thickness after electron irradiation (Fig. 17) can be attributed to a Si supply to the a-Pd–Si layer induced by dissociation of a-SiO_*x*_. Once the alloy composition reaches ∼ 33at%Si, then crystallization would start immediately since the Pd_2_Si is the thermodynamically stable line compound with a remarkably high melting temperature (1604 K) [64] and a large heat of formation (Δ*H *= −43 kJ/mol) [31], as shown in Fig. 18b (location of the Pd_2_Si phase is indicated by a vertical green line) [64]. The liquidus draws a sharp convex parabolic shape with the vertex at 33at%Si, in contrast to the eutectic composition that forms a deep valley around 17at%Si. It is presumed that the supply of Si atoms to the a-Pd-19at%Si layer causes instability of the amorphous phase; i.e. additional solute atom-mediated crystallization, which is a new mechanism of crystallization of amorphous alloy. This reminds us of the crystallization of amorphous nanoparticles by spontaneous alloying, where vapor deposition of Au onto amorphous antimony nanoparticles rapidly formed AuSb_2_ compounds at room temperature [65]. The crystallization accompanied by an abrupt change in chemical composition is similar to that in the present study, except for the difference in morphology.

## Concluding remarks

Electron irradiation effects are classified into knock-on atom displacement and electronic excitation effects, both of which may cause damage to TEM specimens with changes in structure and morphology; hence, there is a widespread understanding that they should be avoided whenever possible. However, there are right interactions between incident electrons and solids that can provide a wealth of valuable insights into elucidating the defects and defect processes in a solid. In this review, we focused on the electronic excitation effect caused by electron irradiation in a TEM and introduced our recent research on solid-state reactions utilizing the dissociation of a-SiO_*x*_ by electronic excitation. The important point is that the process can form metal silicide or crystallize amorphous phase only in the electron-irradiated areas at room temperature or below. Dissociation of oxides by electronic excitation is a highly versatile method for producing metal–metalloid or intermetallic compounds. At present, we have just begun to understand the basic mechanisms of atomic diffusion induced by electronic excitation; however, future investigations will shed light on such effects.

## Data Availability

Data will be made available on request.
